# Variations in psychological disorders, suicidality, and help-seeking behaviour among college students from different academic disciplines

**DOI:** 10.1371/journal.pone.0279618

**Published:** 2022-12-30

**Authors:** Margaret McLafferty, Natasha Brown, John Brady, Jonathon McLaughlin, Rachel McHugh, Caoimhe Ward, Louise McBride, Anthony J. Bjourson, Siobhan M. O’Neill, Colum P. Walsh, Elaine K. Murray

**Affiliations:** 1 Personalised Medicine Centre, School of Medicine, C-TRIC, Altnagelvin Hospital, Ulster University, Derry, Londonderry, United Kingdom; 2 Atlantic Technological University (ATU), Letterkenny, Co. Donegal, Ireland; 3 Western Health and Social Care Trust, Tyrone and Fermanagh Hospital, Omagh, Co. Tyrone, United Kingdom; 4 School of Psychology, Ulster University, Coleraine, Co. Derry, United Kingdom; 5 Genomics Medicine Research Group, School of Biomedical Sciences, Ulster University, Coleraine, Co. Derry, United Kingdom; 6 Centre for Research and Development, Region Gävleborg/Uppsala University, Gavle, Sweden; Chiang Mai University, THAILAND

## Abstract

**Background:**

Elevated levels of suicidality, ADHD, mental ill-health and substance disorders are reported among college students globally, yet few receive treatment. Some faculties and courses appear to have more at-risk students than others. The current study aimed to determine if students commencing college in different academic disciplines were at a heightened risk for psychopathology, substance use disorders and suicidal behaviour, and examined variations in help-seeking behaviour.

**Materials and methods:**

The study utilised data collected from 1,829 first-year undergraduate students as part of the Student Psychological Intervention Trial (SPIT) which commenced in September 2019 across four Ulster University campuses in Northern Ireland and an Institute of Technology, in the North-West of Ireland. The SPIT study is part of the World Mental Health International College Student Initiative (WMH-ICS) which uses the WMH-CIDI to identify 12-month and lifetime disorders.

**Results:**

Students from Life and Health Sciences reported the lowest rates of a range of psychological problems in the year prior to commencing college, while participants studying Arts and Humanities displayed the highest levels (e.g. depression 20.6%; social anxiety 38.8%). However, within faculty variations were found. For example, psychology students reported high rates, while nursing students reported low rates. Variations in help seeking behaviour were also revealed, with male students less likely to seek help.

**Conclusions:**

Detecting specific cohorts at risk of psychological disorders and suicidality is challenging. This study revealed that some academic disciplines have more vulnerable students than others, with many reluctant to seek help for their problems. It is important for educators to be aware of such issues and for colleges to provide information and support to students at risk. Tailored interventions and prevention strategies may be beneficial to address the needs of students from different disciplines.

## Introduction

Internationally, a growing body of literature identifies college students as a group that is particularly vulnerable to psychopathology and suicidal behaviour [1, 2]. Mental health among this population appears to be deteriorating, with college counselling services reporting an increase in students presenting with more severe and complex psychological problems [3–5]. The World Mental Health International College Student Initiative (WMH-ICS) reported that over a third of students from a large, cross-national study presented with at least one clinically significant disorder [6]. The most common problems reported included anxiety, depression, and substance use disorders [6]. Furthermore, comorbidity is common, with between one-fifth and one-quarter of students experiencing multiple disorders [7, 8].

In addition, although research concerning the prevalence of Attention Deficit Hyperactivity Disorder (ADHD) amongst tertiary level students remains scarce, recent literature has indicated prevalence rates ranging from 16% to 27.7% [9, 10]. Concerningly, the presence of ADHD has been directly and indirectly associated with an increased risk of suicidal behaviours among students [10]. Additional concerns relate to the high rates of suicidal behaviours generally, including suicidal ideation, plans and attempts, and self-harm, which are highly prevalent within this population [1, 6, 11, 12], and suicide remains one of the leading causes of death amongst those of college-age [13].

Recent research has indicated that the onset of psychological disorders often occurs prior to the commencement of college [7, 12, 14]. The transition into higher education and adjustment to a less structured learning environment, new social networks and increased academic demands can cause considerable stress and anxiety [15–17] which may exacerbate pre-existing mental illness and correlates with the development of mood disorders in students [18].

The high prevalence of mental illness, suicidality and transitional risk are not the only worrisome trends globally. Research has also revealed rates of treatment-seeking as low as 11%-16% within the student population [14, 19]. The reluctance towards treatment-seeking may be partially attributable to stigma, fears that treatment-seeking will impact future career goals, poor symptom recognition and a preference for self-reliance [7, 20, 21]. Untreated mental illness is a cause for concern, as over time these illnesses can become more severe, treatment-resistant, lead to long-term physical health problems and reduce life satisfaction [22–24].

Within an academic context, untreated mental illness can have a detrimental impact on academic performance, retention, and social connectedness amongst students [20, 25]. As the first onset of mental illness often occurs during the neurodevelopmentally sensitive period between the ages of 18–25, colleges are uniquely positioned to identify at-risk individuals and groups [26]. Early identification may allow for the implementation of tailored and timely mental health and suicide prevention strategies to help ensure students’ academic and psychological welfare.

Detecting specific cohorts at risk of psychological disorders and suicidality is challenging for universities given the vast size and diversity of student populations and the high prevalence of mental illness [6]. Previous research has investigated sociodemographic characteristics as potential risk factors, including gender, age, ethnicity, and sexuality, to aid the early identification of vulnerable sub-groups [7, 11, 27]. Subsequently, some colleges have implemented more targeted and informed strategies to encourage help-seeking amongst these groups, and many offer more socially and culturally inclusive support services. However, more evidence-based solutions are required to further engage students and improve help-seeking behaviours.

A key, yet frequently overlooked method of risk detection, may arise from exploring mental illness and help-seeking prevalence within different academic disciplines. Although there remains a paucity of research exploring the relationship between academic disciplines and psychological disorders, preliminary findings suggest that specific degree courses may attract individuals who are more susceptible to mental illness [28–30].

For example, a large, cross-sectional study spanning 81 American universities found that students studying art and design presented with the highest rates of mental illness [30]. Almost 45% of art and design students reported at least one disorder, followed closely by humanities (39%). Art and humanities students also had the highest rates of suicidal ideation and over one fifth of students from these disciplines reported having engaged in self-injury. Disciplines demonstrating the lowest rates of mental illness included engineering (31%), public health (28%) nursing (28%) and business (27%). Likewise, a recent study conducted reported that students from arts and humanities, social work, and behavioural, and social sciences, were more likely to report emotional and substance use disorders in comparison to their peers from business or engineering disciplines [31]. An Australian study also indicated that students studying in non-health professional courses had significantly higher levels of psychological distress, with law students (58%) demonstrating the highest rates [19]. This is in line with recent research which reported that law students had the poorest mental wellbeing and some of the highest rates of lifetime suicide attempts (8.4%) [29].

However, research findings are mixed, as an Irish study revealed that business students were more likely to abuse alcohol than medical students [32]. Another study also reported higher rates of depression and alcohol use among business students when compared to medical students [33]. Furthermore, in contrast to a study by Lipson and colleagues [30], very high rates of distress were demonstrated amongst engineering students, with over half experiencing significant psychological distress (52%). Worryingly, these students also had the lowest levels of help-seeking, with only 6% having sought treatment [19]. This may be related to a high prevalence of males engaging in engineering courses, since prior research has revealed that males are less likely to seek help for emotional problems [7]. Lipson and colleagues [30] also reported a low prevalence of help-seeking (20%) among engineering students but a higher uptake among those taking mental health related courses. A recent study corroborates these findings, with students from engineering or business courses less likely to attend counselling [34].

Many of the previous studies investigating mental health risks among college students in specific degree courses utilise second-and third-year students and graduate students in study samples [19, 29, 31]. Subsequently, this creates difficulties in deciphering whether specific students present with heightened susceptibility to mental illness upon college entry or whether stressors relating to specific courses result in the development of psychological problems, therefore it is important to study students as they commence college for the first time. Furthermore, many studies, which have explored mental health among various college disciplines, have predominantly focused on healthcare professions, while disciplines relating to art, humanities, social sciences, and computing have been studied much less frequently [30]. As many students from a wide range of disciplines present with mental health difficulties during their time at college, it is important to include students from various disciplines, in order to help identify those most at risk, which may lead to tailored support for these cohorts within the academic setting. Moreover, the measures typically used in other studies focus predominantly on current anxiety, depression and psychological distress, rather than disorders in the time prior to commencing college.

This study aims to addresses the above concerns and extends upon previous findings by; 1) exploring a broader range of academic disciplines and faculties, with the prevalence of a wide range of mental health and substance disorders, ADHD, and suicidal behaviour in the year prior to commencing college explored using validated, clinical measures and2) examining help seeking behaviour among this cohort. Based on previous research, it is hypothesised that students within the arts, humanities and social science faculties will demonstrate the highest prevalence of mental illness. Additionally, it is hypothesised that help-seeking may be lower in male dominated degree courses, as previous studies have shown that many males are reluctant to seek help for a mental health problem.

## Materials and methods

### Design

In September 2019, the Student Psychological Intervention Trial (SPIT), commenced across five sites split between Ulster University (UU), Northern Ireland (NI), and an Institute of Technology (IT)in the North-West of the Republic of Ireland (ROI). Funded by the Cross-border Healthcare Intervention Trials in Ireland Network (CHITIN), the SPIT study, was conducted as part of the World Mental Health International College Student Initiative (WMH-ICS). The aim of the WMH-ICS is to conduct global research into the mental health and wellbeing of students during their time at college utilising a longitudinal cohort design, with participants completing the comprehensive baseline survey, developed by the WMH-ICS, when they first start college life. (Follow up studies are conducted when students commence their second and third year at college). The SPIT study obtained ethical approval from the Ulster University Research Ethics Committee (REC/19/0072).

### Sample

All first-year undergraduate students at UU and first year students from a number of courses at the IT received an email inviting them to consider taking part in the SPIT study prior to registering with the university/college. A participant information sheet was attached, along with contact details for the researchers should they require further information. Researchers addressed students during welcome meetings when they first arrived on campus and invited them to participate. Trained researchers obtained written informed consent from all participants. Overall, 1,947 students consented and were given a unique ID number. The students entered only their ID number at the start of the online survey, to ensure survey responses was anonymous. The baseline survey was fully completed by 1,829 participants and they received a college branded sweatshirt to thank them for their participation. In accordance with WMH-ICS guidelines and so that accurate comparisons can be made with other WMH-ICS surveys, international students, those under the age of 18 and students who were repeating first year were excluded from the study.

### Discipline/faculty breakdown

Ulster University (Campuses B-E) consists of four disciplines, known as faculties, with a number of different courses embedded in each faculty. The IT (Campus A) has a similar faculty and course structure. See Table 1 for details on the courses provided within each faculty and the breakdown of participants per faculty on each campus, including a gender breakdown per campus. The majority of student participants from Campus A and Campus C were from the faculty of Life and Health Sciences. Campus D is the largest of the UU campuses, with the highest percentage of male students. The majority of participants based in Campus D were from either the faculty of computing, engineering and the built environment or the faculty of arts, humanities and social sciences. The majority of students based on Campus E were from the faculty of Life and Health Sciences. Campus B has predominately students in the arts, humanities and social sciences faculty.

**Table 1:** Breakdown of faculties, courses and number of participants per faculty.

Faculty	Main courses	Participants per campus
**Life and Health Sciences** (n = 806)	Nursing, mental health nursing, psychology, food nutrition, biomedical science, stratified medicine, podiatry, pharmacy, sport, geography, childcare	A (230; m 43, f 188)
		C (219; m 30, f 189)
		D (73; m 17, f 56)
		E (281; m 22, f 269)
**Business School** (n = 245)	Business, accountancy, economics, communication, HR, leisure, tourism	A (30; m 13, f 18)
		B (18; m 4, f 14)
		C (50; m 8, f 189)
		D (74; m 24, f 56)
		E (71; m 28, f 43)
**Arts, Humanities and Social Sciences** (n = 509)	Art, drama, Irish, English, history, social policy, social work, youth work	A (49; m 16, f 33)
		B (150; m 33, f 117)
		C (31; m 11, f 20)
		D (119; m 24, f 95)
		E (157; m 45, f 112)
**Computing, Engineering and the Built Environment** (n = 258)	Computing, technology, architecture, construction, engineering	A (50; m 39, f 11)
		B (17; m 9, f 8)
		D (103; m 70, f 33)
		E (88; m 73, f 15)

Note: m = male, f = female

### Diagnostic assessment

The comprehensive online survey which examines the prevalence of mental health disorders in accordance with DSM-V criteria, was hosted on Qualtrics software. Questions for the survey were adapted from the WMH-Composite International Diagnostic Interview Screening Scales (WMH-CIDI -SC) [35, 36] by the WMH-ICS team. Good concordance has been reported between the CIDI-SC and clinical assessment, with Area Under the Curve (AUC) in the range of 0.70–0.78 [36]. The survey enquires about a range of mental health problems (for example, mood, panic disorders, bipolar disorder), ADHD, and substance related disorders, within the past 12-months. Lifetime disorders are also assessed, as well as age of onset. The Self Injurious Thoughts and Behaviour Interview, (SITBI) was utilised to identify students with suicidal thoughts, plans and attempts [37], with 15 questions related to suicidal behaviour included in the survey. The SITBI has been found to have strong interrater reliability and test-retest reliability over a period of 6 months, and concurrent validity has been revealed between the instrument and other measures of suicidal behaviour [39].

### Help seeking

The WMH-CIDI includes a number of questions related to help-seeking behaviour. Participants were asked if they ever received psychological counselling or if they received medication for an emotional or substance use problem, and if so, were they still in treatment. Those not in treatment were asked if there was ever a time in the previous 12 months when they felt that they may have needed help for an emotional or substance use problem. Subsequent questions enquired about reasons for not seeking help, rated on a 5-point Likert scale, ranging from unimportant to very important in order to ascertain barriers to help seeking behaviour.

### Data analyses

The prevalence rates of a range of mental health and substance abuse problems and suicidal behaviour reported on each campus were examined. The demographic characteristics of the student participants in each of the four faculties were explored and comparisons made with regards to the prevalence of disorders reported within the faculties and help seeking behaviour during the previous 12 months. Further analyses explored the prominence of mental health disorders in the eight most populated courses and their willingness to get help for a mental health or substance problem. Chi square tests of independence were used to identify significant variations in prevalence rates. Note, with regards to missing values, 9 students failed to include details related to their faculty, course or campus and had to be omitted from some parts of the analyses. Weights were applied to all analyses to ensure that the findings could be generalised to the student populations in both institutions in relation to age and gender.

## Results

The breakdown of mental health and substance abuse problems, ADHD, and suicidal behaviour in the year prior to starting university were reported on each of the campuses are shown in Table 2. Students on the Campus B reported the highest rates of depression (18.7%, panic disorder (12.6%), bipolar disorder (4.2%) and social anxiety (44.3%). Campus A student participants had the highest rates of alcohol and drug abuse and suicidal behaviour. Students on the Campus C reported the highest rates of self-harm (8.4%). Students on Campus D had the lowest rates of depression and suicidal behaviour. Students in Campus E reported the lowest rates of panic or bipolar disorders, social anxiety and self-harm. Chi-square tests of independence revealed significant inter-campus differences in reported rates for panic disorder, social anxiety, suicide ideation and suicide plans and self-harm. While variations were revealed across campuses, further analyses were conducted to explore if these differences were related to the demographic characteristics of the students, and the faculties they were enrolled in.

**Table 2:** Prevalence of 12-month mental health problems, ADHD, substance abuse and suicidal behaviour per campus.

Total	Campus A		Campus B		Campus C		Campus D		Campus E		
	360		185		303		375		597		
	n	*%*	n	*%*	n	*%*	n	*%*	n	*%*	χ 2
Depression	64	*18*.*5*	36	*18*.*7*	46	*14*.*1*	47	*11*.*8*	73	*12*.*9*	11.771
Panic Disorder	**26**	***6*.*6***	**27**	***12*.*6***	**29**	***9*.*0***	**26**	***5*.*9***	**37**	***5*.*6***	**13.398** *
Any Bipolar	12	*3*.*5*	7	*4*.*2*	9	*3*.*4*	12	*3*.*3*	16	*2*.*7*	1.824
Social Anxiety	**124**	***34*.*1***	**82**	***44*.*3***	**104**	***34*.*9***	**111**	***29*.*0***	**153**	***24*.*2***	**35.057** ***
ADHD	111	*31*.*8*	64	*31*.*5*	80	*27*.*1*	97	*26*.*2*	147	*23*.*7*	12.129
Alcohol abuse	47	*13*.*2*	15	*8*.*4*	26	*9*.*7*	43	*12*.*0*	68	*11*.*1*	5.438
Drug abuse	28	*7*.*9*	10	*4*.*9*	8	*4*.*1*	24	*7*.*8*	28	*4*.*9*	8.664
Suicide Ideation	**78**	***22*.*9***	**42**	***20*.*1***	**55**	***18*.*0***	**51**	***13*.*1***	**94**	***15*.*6***	**17.182** **
Suicide plan	**41**	***12*.*1***	**16**	***7*.*4***	**22**	***7*.*4***	**20**	***5*.*4***	**34**	***6*.*1***	**15.171** *
Suicide attempt	8	*2*.*1*	8	*3*.*7*	4	*1*.*1*	7	*1*.*7*	11	*1*.*9*	3.360
Self-harm	**26**	***7*.*1***	**10**	***4*.*1***	**25**	***8*.*4***	**17**	***4*.*0***	**19**	***3*.*1***	**16.604** *

Note: n = unweighted, % = weighted, χ 2 tests show significant differences in prevalence rates

*p < .05

**p < .01

***p < .001

Further analyses compared differences between faculties in relation to demographics and prevalence rates of disorders. Table 3 shows the demographic characteristics of student participants from each faculty across all campuses (N = 1,829), as previous research has revealed age, gender and sexuality differences in relation to psychopathology and suicidality. Students in L&HS were most likely to be female (77.5%) and aged 21 or older (35.9%) than those in other faculties. In the Business School, students were most likely to be younger (81.5%). Students in the Faculty of AH&SS were more likely to identify as non-heterosexual (17.9%) than those in the other faculties. The highest number of male students can be found in the Faculty of CE&BE (84.7%).

**Table 3:** Demographic characteristics of student participants from each faculty.

Total	Life and Health Sciences (L&HS)		Business School		Arts, Humanities and Social Sciences (AH&SS)		Computing, Engineering and the Built Environment (CE&BE)		
	806		245		509		258		
	n	*%*	n	*%*	n	*%*	n	*%*	χ 2
**Gender**									
Male	111	*22*.*5*	76	*48*.*1*	129	*41*.*3*	191	*84*.*7*	**377.478** ***
Female	695	*77*.*5*	169	*51*.*9*	380	*58*.*7*	67	*15*.*3*	
**Age**									
Under 21	588	*64*.*1*	210	*81*.*5*	420	*75*.*5*	192	*69*.*1*	**34.412** ***
21 and over	218	*35*.*9*	35	*18*.*5*	89	*24*.*5*	66	*30*.*9*	
**Sexuality**									
Heterosexual	732	*91*.*3*	222	*91*.*3*	409	*82*.*1*	224	*88*.*3*	**26.808** ***
Non-Heterosexual	69	*8*.*7*	22	*8*.*7*	97	*17*.*9*	34	*11*.*7*	

Note: n = unweighted, % = weighted, χ 2 tests show significant differences in prevalence rates

*p < .05

**p < .01

***p < .001

The prevalence rates, per faculty, of various mental health problems and suicidal behaviour, along with details of those currently receiving treatment, or felt that they may have needed treatment in the past 12 months are shown in Table 4. Chi-square tests of independence revealed significant differences between faculties in reported rates for depression, panic disorder, bi-polar disorder, social anxiety, possible ADHD, alcohol and drug abuse and self-harm. No significant differences were found between faculties for suicidal ideation plans or attempts or for currently receiving treatment. Significant variations were revealed however between faculties with regards to feeling that they may have need treatment in the previous 12 months.

**Table 4:** Prevalence of 12-month mental health problems, ADHD, substance abuse, suicidal behaviour and help seeking reported by students from each faculty.

	Total		Life and Health Sciences (L&HS)		Business School		Arts, Humanities and Social Sciences (AH&SS)		Computing, Engineering and the Built Environment(CE&BE)		
Participants	1829		806		245		509		258		
	n	*%*	n	*%*	n	*%*	n	*%*	n	*%*	χ 2
Depression	**268**	***14*.*5***	**93**	***11*.*5***	**30**	***13*.*5***	**108**	***20*.*6***	**34**	***13*.*2***	**20.223** ***
Panic Disorder	**145**	***7*.*0***	**61**	***7*.*1***	**19**	***7*.*1***	**53**	***9*.*7***	**10**	***2*.*8***	**14.340** **
Any Bipolar	**84**	***4*.*6***	**25**	***3*.*2***	**12**	***5*.*4***	**38**	***7*.*2***	**8**	***3*.*6***	**13.197** **
Social Anxiety	**576**	***30*.*6***	**223**	***26*.*5***	**79**	***30*.*7***	**201**	***38*.*8***	**72**	***28*.*7***	**25.825** ***
ADHD	**507**	***27*.*2***	**193**	***24*.*0***	**65**	***26*.*0***	**176**	***34*.*5***	**65**	***24*.*9***	**18.273** **
Alcohol abuse	**201**	***11*.*3***	**64**	***7*.*8***	**38**	***16*.*6***	**67**	***14*.*5***	**30**	***10*.*5***	**20.921** ***
Drug abuse	**98**	***6*.*0***	**27**	***4*.*0***	**17**	***7*.*6***	**37**	***7*.*8***	**17**	***6*.*6***	**10.691** *
Suicide Ideation	320	*17*.*2*	118	*15*.*0*	43	*16*.*6*	112	*21*.*2*	46	*17*.*2*	8.934
Suicide plan	133	*7*.*4*	46	*6*.*2*	16	*6*.*5*	52	*9*.*7*	19	*7*.*8*	6.813
Suicide attempt	38	*1*.*9*	11	*1*.*5*	4	*2*.*0*	20	*3*.*3*	3	*1*.*2*	6.970
Self-harm	97	*4*.*9*	40	*4*.*8*	11	*4*.*6*	35	*6*.*0*	11	*3*.*9*	2.906
Currently in treatment	103	*5*.*4*	37	*4*.*7*	10	*5*.*7*	42	*7*.*4*	13	*5*.*3*	3.242
Felt they may have needed treatment	**191**	***11*.*3***	**82**	***10*.*4***	**22**	***11*.*5***	**68**	***16*.*3***	**19**	***7*.*3***	**16.544** ***

Note: n = unweighted, % = weighted, χ 2 tests show significant differences in prevalence rates

*p < .05

**p < .01

***p < .001

When comparing the prevalence rate of mental health and substance disorders and suicidal behaviour, students in the faculty of L&HS were least likely to have depression (11.5%), any bipolar disorder (3.2%) and social anxiety (26.5%). They were also least likely to have alcohol use disorder or drug abuse and dependence. Students in the Business School reported the highest rate of 12-month alcohol use at 16.6%. Those in faculty of AH&SS were most likely to display the highest rates of mental health disorders, ADHD, drug abuse or drug dependence. Students in the faculty of CE&BE were least likely to have panic disorder (2.8%) or engage in self-harm (3.9%). Students in the faculty of AH&SS were most likely to be currently receiving treatment for a mental health problem (although not statistically significant), but the overall number was low in comparison to the prevalence of disorders, with only 7.4% currently receiving either psychological counselling or taking medication for their problem. However, 16.3% felt that they may have needed help for their problems in the previous 12 months. Student participants from CE&BE were least likely to indicate that they may have needed help in the 12 months prior to commencing college (7.3%).

As these faculties include a wide range of courses, as shown previously in Table 1, especially the faculty of L&HS, further analyses were conducted to establish if there were differences in prevalence rates of disorders and treatment uptake within the faculties. The eight courses with the highest number of students were selected as shown in Table 5.

**Table 5:** Prevalence of 12-month mental health problems, ADHD, substance abuse, suicidal behaviour and help seeking reported by students from different courses.

	Total		Psychology		Mental Health Nursing		Nursing		Business		Art		Law		Engineering		Computing		
Participants	1829		84		65		255		99		144		84		83		135		
	n	*%*	n	*%*	n	*%*	n	*%*	n	*%*	n	*%*	n	*%*	n	*%*	n	*%*	χ 2
Depression	**268**	***14*.*5***	**19**	***19*.*4***	**4**	***5*.*6***	**24**	***10*.*2***	**13**	***11*.*9***	**30**	***19*.*6***	**15**	***17*.*4***	**7**	***8*.*8***	**24**	***18*.*0***	**18.902** **
Panic Disorder	**145**	***7*.*0***	**16**	***17*.*7***	**7**	***11*.*1***	**7**	***7*.*7***	**8**	***8*.*4***	**19**	***11*.*8***	**9**	***10*.*6***	**1**	***0*.*6***	**7**	***3*.*7***	**41.170** ***
Any Bipolar	**84**	***4*.*6***	**5**	***6*.*0***	**3**	***3*.*9***	**4**	***1*.*5***	**5**	***5*.*1***	**12**	***8*.*3***	**3**	***3*.*8***	**0**	***0***	**7**	***5*.*4***	**16.729** *
Social Anxiety	**576**	***30*.*6***	**40**	***50*.*5***	**18**	***24*.*4***	**46**	***16*.*1***	**33**	***31*.*7***	**66**	***46*.*5***	**28**	***31*.*7***	**19**	***24*.*5***	**41**	***30*.*9***	**53.699** ***
ADHD	**502**	***27*.*2***	**27**	***33*.*3***	**18**	***28*.*6***	**53**	***19*.*8***	**24**	***21*.*8***	**53**	***34*.*9***	**27**	***32*.*1***	**18**	***19*.*6***	**37**	***27*.*9***	**17.586** **
Alcohol abuse	201	*11*.*3*	4	*6*.*6*	7	*10*.*2*	20	*6*.*5*	14	*15*.*3*	11	*8*.*4*	13	*16*.*1*	9	*11*.*0*	15	*9*.*8*	11.842
Drug abuse	98	*6*.*0*	3	*8*.*0*	3	*5*.*3*	7	*2*.*3*	10	*10*.*5*	10	*6*.*6*	6	*8*.*7*	6	*8*.*1*	10	*6*.*6*	12.329
Suicide Ideation	**320**	***17*.*2***	**19**	***22*.*3***	**10**	***15*.*7***	**27**	***11*.*5***	**18**	***15*.*5***	**34**	***20*.*3***	**21**	***25*.*8***	**14**	***15*.*0***	**26**	***20*.*2***	**14.691** *
Suicide plan	**133**	***7*.*4***	**7**	***9*.*5***	**5**	***7*.*8***	**8**	***3*.*9***	**5**	***4*.*3***	**14**	***8*.*1***	**12**	***14*.*9***	**6**	***7*.*3***	**12**	***9*.*5***	**14.591** *
Suicide attempt	38	*1*.*9*	2	*2*.*1*	2	*3*.*5*	2	*1*.*4*	1	*0*.*8*	8	*4*.*9*	4	*3*.*9*	0	*0*	3	*2*.*2*	9.955
Self-harm	97	*4*.*9*	5	*4*.*9*	2	*3*.*5*	3	*1*.*2*	6	*5*.*0*	10	*5*.*4*	3	*3*.*9*	4	*3*.*8*	6	*4*.*4*	6.998
Currently in treatment	**103**	***5*.*4***	**9**	***11*.*1***	**6**	***11*.*3***	**5**	***2*.*1***	**6**	***5*.*2***	**19**	***11*.*0***	**4**	***4*.*8***	**3**	***2*.*6***	**10**	***8*.*4***	**19.277** **
Felt they may have needed treatment	**191**	***11*.*3***	**16**	***21*.*7***	**5**	***7*.*7***	**21**	***7*.*9***	**9**	***10*.*9***	**19**	***17*.*6***	**12**	***16*.*5***	**1**	***1*.*3***	**14**	***10*.*3***	**29.174** ***

Note: n = unweighted, % = weighted, χ 2 tests show significant differences in prevalence rates

*p < .05

**p < .01

***p < .001

Chi-square tests of independence revealed significant differences between courses in reported rates for depression, panic disorder, bi-polar disorder, social anxiety, possible ADHD and suicidal ideation and plans and help seeking. No significant differences were found between courses for alcohol misuse, drug abuse or suicide attempts or self-harm. Students from the School of Psychology reported elevated rates of a range of mental health problems and suicidal behaviour, with particularly high rates of panic disorder (17.7%) and social anxiety (50.5%). Students in the School of Nursing generally reported low rates of all disorders and suicidal behaviour. Mental health nursing students had the lowest rates of depression (5.6%), but the prevalence rates of other disorders were higher than the rates found in general nursing. Art students had the highest prevalence of possible ADHD (34.9%) and depression (19.6%). Law students were most likely to endorse suicidal ideation or plan. Engineering students reported low rates of mental health problems and suicidal behaviour, with particularly low rates of panic disorder and no suicide attempts reported. Computing students reported rates of depression and suicidal behaviour that were higher than average.

In relation to getting help for an emotional or substance use problem, 23.1% of participants reported that they had received psychological counselling and 13.1% reported that they had taken medication. The average age that participants received help for the first time was 17. Only 103 participants were currently in treatment. Students from psychology, mental health nursing and art were most likely to be receiving treatment for a mental health problem, while nursing students were least likely (2.1%), followed by engineering students (2.6%) as shown in Table 5. All participants who indicated that they hadn’t received any treatment in the previous 12 months were asked if there was ever a time in the past 12 months when they felt that they might need psychological counselling or medication for any emotional or substance use problems. Overall, 191 students (11.3%) indicated that they thought they may have needed help. Psychology students were the most likely to say that they felt that they needed help for their problems (21.7%), while only one engineering student felt they needed help as shown in Table 5.

The participants who felt they may have needed help were subsequently asked how important they rated various reasons for why they did not seek help as outlined in Table 6. The most important reasons were that they wanted to handle it themselves (34.1%) or they talked to friends or family (24.4%). The reasons of least importance were related to costs, problems with time or transportation and being afraid of it impacting on their school or professional career.

**Table 6:** Reasons for not seeking help for a mental health or substance problem.

Total 191	Very important		Important		Moderately important		Of little importance		Unimportant	
	n	%	n	%	n	%	n	%	n	%
Not sure if available treatments were effective	16	*9*.*1*	27	*13*.*1*	48	*27*.*3*	33	*16*.*3*	66	*34*.*2*
Wanted to handle the problem on their own	65	*34*.*1*	51	*27*.*5*	43	*23*.*7*	17	*8*.*3*	14	*6*.*3*
Too embarrassed	36	*17*.*7*	48	*23*.*4*	36	*19*.*2*	34	*19*.*0*	35	*20*.*7*
Talked to friends or family instead	50	*24*.*4*	53	*28*.*9*	36	*18*.*9*	25	*13*.*1*	26	*14*.*6*
Costs too much money	23	*11*.*2*	20	*10*.*1*	25	*13*.*0*	41	*23*.*6*	80	*42*.*1*
Unsure of where to go or who to see	41	*20*.*5*	32	*17*.*5*	37	*19*.*3*	34	*19*.*1*	44	*23*.*6*
Problems with time, transportation or scheduling	21	*10*.*1*	26	*13*.*2*	27	*13*.*4*	38	*21*.*4*	77	*42*.*0*
Afraid it might harm your school or professional career	24	*12*.*6*	28	*14*.*3*	26	11.8	40	*22*.*2*	69	*39*.*1*
Worried that people might treat you differently	30	*15*.*4*	45	*22*.*3*	35	18.2	32	*16*.*5*	45	*27*.*5*

Note: n = unweighted, % = weighted

Of particular interest, further analyses revealed that students on computing courses were least likely to rate that seeking help might do harm to their college or professional career (9.3%). However, a high percentage of psychology (22.9%) nursing (22.8%), mental health nursing (20.7%) and law (20.3%) students rated it as being very important.

## Discussion

Early analysis of the SPIT data revealed variations in the prevalence of mental health and substance disorders, suicidal behaviour and ADHD among undergraduate students commencing college in NI and the ROI [10, 12]. The current study added to this research, revealing that prevalence rates varied between faculties and also within faculties, providing important information for educators, with some courses having more students with psychological difficulties than others. Variations in help seeking behaviour were also revealed.

Overall, the study found high prevalence rates of suicidal behaviour and a range of mental health and substance abuse problems, which is of great concern. Suicidal behaviour was particularly high among students in the IT in the ROI. A recent study conducted among third level students in Ireland, found that students attending Institutes of Technology had poorer mental health [38]. Furthermore, the IT has the highest proportion of students from a disadvantaged area, when compared to other higher education institutes in the ROI [39], which may partially explain the elevated prevalence rates revealed in the current study.

When examining students attending college in NI, variations were revealed across the four UU campuses, with very high rates of psychopathology revealed among those attending Campus B. Conversely, students on Campus D reported the lowest rates of mental health problems but had the highest rate for drug abuse, and when comparing students across the NI campuses they also had the highest rate for alcohol abuse. Some geographical locations may have higher levels of mental health problems in the population than others for a number of reasons, for example, they may have elevated levels of deprivation. However, as students are not necessarily from the area the campuses are located in, further analyses were conducted to determine why these variations might occur.

The finding that students on Campus D were the least likely to engage in suicidal behaviour and have the lowest levels of depression or panic disorder, but high rates of substance abuse may be related to the demographic characteristics of students attending the campus, with males making up a large percentage of the student population. This is in line with previous research which reported higher levels of substance problems among male students, while females are more likely to have internalizing problems such as depression [7], as was found in the current study. It should also be noted however, that males may be less likely to admit to having a psychological problem, due to stigma, internalized traditional masculine norms and fears of looking weak [40]. Indeed, studies have found that young males are more prone to underreporting symptoms of mood disorders, and demonstrate higher levels of denial about psychological disorders on self-report surveys [41, 42].

When considering variations in prevalence rates between faculties, the lowest rates of mental health and substance problems were revealed among students in L&HS. A higher proportion of students in L&HS were 21 or over when compared to students from other faculties. Many students may have completed exams in the year prior to starting college, which may partially account for the elevated 12-month prevalence rates reported among younger students. The highest prevalence rates were found within the faculty of AH&SS, with the exception of alcohol misuse which was highest amongst students from the Business School. This is consistent with findings from another Irish study [32]. It should be noted that the Business School had the highest percentage of younger students in the current study, therefore this finding is particularly concerning, as prolonged, heavy alcohol usage throughout the academic course of study is associated with poorer academic performance, attrition, and increased likelihood of mood disorders [43]. Substance awareness campaigns during secondary education may be beneficial and further information sessions in the college setting would be advantageous.

In relation to treatment seeking, students in L&HS were least likely to have received treatment while those in AH&SS were most likely to have received treatment or felt that they may have needed treatment in the year prior to starting college, which is in line with the reported prevalence rates. Nevertheless, the proportion of students receiving or acknowledging that they may need help is much lower than the prevalence rates of disorders reported. In accordance with prior studies [34], students from CE&BE were least likely to believe that they may have needed help, which may be related to the fact that many males are enrolled on these courses. Indeed, it has been reported that male college students tend to have poorer mental health literacy and are poorer at recognising symptoms of depression, therefore male dominated courses may have lower levels of help-seeking and they may score lower on self-report questionnaires due to poor recognition of symptoms [44]. The most important reason participants in the current study reported for not seeking help was that they wanted to handle it themselves or they talked to friends or family. It is important therefore to encourage help seeking behaviour and provide information on a wide range of services available within the college and community setting.

Further analyses were conducted to uncover at risk subgroups, within the student population. While many students in the faculty of L&HS had low levels of mental health problems, when individual courses were examined, distinct variations were uncovered. For example, psychology students reported elevated rates of panic disorder and social anxiety, which is in line with previous research [20]. Furthermore, these students were most likely to say that they felt that they may have needed treatment which would suggest that while the psychology students had an awareness about their mental health issues, it did not encourage them to seek help. This is in accordance with prior research which reported that taking psychology classes does not appear to predict positive attitudes to seeking mental health care for mental health problems [45, 46]. One of the reasons provided for not seeking help, by psychology students in the current study, was a fear of it impacting on their academic and future career. These findings would imply that when promoting help-seeking behavior among those in the health profession, especially psychology students, it is important to be mindful of their fears and encourage them to disclose any problems.

Nursing students were least likely to report a range of psychological problems. It should be noted however that mental health nursing students reported higher rates of disorders than those studying general nursing, with the exception of depression, which may have drawn them towards the course, in the first instance. Mental health nursing students were most likely to have received treatment despite lower levels of disorders. This may be related to their knowledge and understanding of the importance of treatment [20]. Engineering students were least likely to have received treatment or felt that they needed treatment, but they also reported low levels of psychological and substance related issues. As this is a male dominated course, the question remains if these findings may be related to a reluctance of males to disclose mental health issues and to seek help for their problems or poor symptom awareness.

Indeed, many students appear to be unwilling to seek help from traditional sources within the college. This maybe be because they are concerned that the information may be shared with academic staff, although if adjustments are made, such as extensions to deadlines, this can be very advantageous. Moreover, stigma, preference for self-management and time commitments can be deterrents. Students on the health professional courses, for example, mental health nurses or psychology students, may be particularly reluctant to divulge that they have a mental health problem for fear that it may impact on their career [46]. It may be useful therefore for colleges to employ the use of more anonymised, self-directed, digital or online interventions [47]. The second phase of the SPIT study involves the trial of an online CBT based guided intervention and the findings may provide a useful alternative to face-to-face sessions, particularly for those who don’t want to seek help from traditional sources.

The study also found that art students, in particular, reported very high rates of a range of mental health problems and suicidal behaviour, which is in accordance with prior research [30]. An interesting finding was that the prevalence rate of bipolar disorder among those studying art was almost twice the average rate reported in the study overall. Indeed, studies have revealed high levels of creativity among those with bi-polar disorder [48, 49]. Almost a fifth of art students also had clinical levels of depression in the previous 12 months and were most likely to engage in self-harm or have attempted suicide. It is very important therefore that support is offered to this vulnerable cohort within the college setting.

Business students reported the highest rate of drug abuse, while law students reported the highest alcohol misuse rates and suicide ideation and plans but not attempts. Prior studies have suggested that such findings are connected to stressors related to the course [31, 50]. It must be remembered, however, that this cohort was surveyed shortly after registering at college, before they had engaged on their course. It may be beneficial to conduct further research to help determine factors that may draw students with such problems towards these courses.

Previous authors have theorised that specific traits and environmental influences shape intellectual interests and attract certain individuals to specific courses [51]. Socioeconomic status and problematic early life experiences may have an impact. Widening access to higher education, while being extremely beneficial, can bring additional challenges, with students enrolling from diverse backgrounds. They may be attracted towards certain courses, such as psychology or law, due to negative early life experiences. For example, it has been found that students who study humanities, social work and counselling were more likely to report childhood adversities [52, 53], which are strongly associated with poor mental health [11]. Additionally, personality is thought to influence degree selection. For example, high levels of neuroticism have been found in law and psychology students [29, 54]. These factors may not only attract individuals towards specific degrees but also predispose them to poorer mental wellbeing. Further research is therefore warranted to explore these risk factors in greater detail and plans are in place to conduct additional analyses utilising the SPIT data.

As this study identified courses with many at-risk students it may be beneficial to provide targeted support and information to students through their lectures and encourage social interaction with their peers, creating a sense of belonging as they embark on their college life. Discipline specific support may be warranted. For example, students on some courses, such as those involving the arts, may feel isolated and they may benefit from initiatives to increase social interaction. Peer Assisted Study Sessions (PASS) and wellbeing sessions may be beneficial to help support the transition to university life [55]. As these are embedded within courses, such sessions can be tailored to the needs of the students, addressing the individual challenges of that specific course, with first year students being supported by their higher year peers.

### Limitations

While this study identified several important findings, a number of limitations should be considered when interpreting the results. Firstly, the study relies on self-report measures, therefore some students may not have responded accurately or honestly. Secondly, only the 191 students who said that they felt that they may have needed help in the previous year, but were not in treatment, were asked the subsequent question related to reasons why they would not seek help, therefore these findings may not be generalised to the wider population. Furthermore, the current study is cross-sectional in nature, and is based on findings from year one only, when students started college for the first time. It will be very beneficial to monitor these students throughout their time at college, to determine if suicidality, psychopathology and substance abuse prevalence rates vary as they progress through their courses, some of which may be more stressful and academically challenging than others. Finally, it should be noted that a number of courses were not well represented in the study.

To conclude, the study revealed that many students commence college with pre-existing psychological and substance related problems and suicidal behaviour. However, the prevalence rates varied considerably across academic disciplines, with some courses having many at-risk students enrolled. However, many of these students did not seek help for these problems. It is important therefore for educators to be aware of such issues and for colleges to provide information and support to students at risk. Tailored interventions and prevention strategies may be beneficial to address the needs of students from different disciplines. This may be even more important, since the pandemic, when students were working remotely, with some cohorts missing out on practical classes, lab work and placements, with many struggling since the return to face-to face leaning. The findings from this study should appeal to educators and those with an interest in student mental health and wellbeing.
